# Trajectories of activities of daily living according to dementia among middle-aged and older people in South Korea: a longitudinal study from 2006 to 2020 (14 years)

**DOI:** 10.3389/fpsyt.2024.1356124

**Published:** 2024-05-15

**Authors:** Soo Eun Chae

**Affiliations:** Department of Education, Art and Humanities College, Gangneung–Wonju National University, Gangneung-si, Republic of Korea

**Keywords:** activities of daily living (ADL), instrumental activities of daily living (IADL), dementia in older adults, latent growth curve models, Korea longitudinal study of ageing (KLoSA)

## Abstract

**Introduction:**

The aging population in South Korea faces numerous health challenges, one of which is the decline in Activities of Daily Living (ADL) and Instrumental Activities of Daily Living (IADL). This study aims to investigate the patterns of change in ADL and IADL among older adults and examines how these patterns vary between individuals with and without dementia.

**Methods:**

We conducted an analysis of data collected from the Korea Longitudinal Study of Ageing (KLoSA) between 2006 and 2022. Our cohort consisted of individuals aged 45 and older with non-dementia conditions, including mild cognitive impairment (*N*=6042), and a smaller group with dementia (*N*=91). Using Latent Growth Curve Models, we explored the developmental trajectories of ADL and IADL among our sample.

**Results:**

Our findings indicate a linear decline in both ADL and IADL scores as individuals age. The decline in IADL was more pronounced in the dementia group, suggesting a greater sensitivity to sociocultural factors within this domain. The data revealed that individuals with dementia had consistently lower ADL and IADL scores. Notably, the variance in scores within the dementia group increased with age, signifying a worsening in daily living performance and an increase in individual variation (*F*=226.630, *p*<.001).

**Discussion:**

The results of this study underscore the impact of dementia on both the self-regulation function and the social and cultural aspects of daily living performance, particularly reflected in IADL scores. These findings point to the necessity for comprehensive care strategies that address the multifaceted needs of older adults with dementia, including support for complex daily activities that are influenced by sociocultural factors.

## Introduction

As South Korea transitions into an aging society, the necessity for preparedness against various age-related disorders escalates. Consistent with prior research findings, aging in humans is associated with a spectrum of declines, encompassing physical and functional deterioration, weight loss, and diminished physical activity (1–3). This decline is an ongoing process, not confined to specific periods or types, affecting each individual progressively (2). The aging process manifests diverse patterns and factors of frailty. Notably, cognitive decline in older adults is a significant aspect of aging. This cognitive decline, often concomitant with aging, is frequently linked with neurocognitive disorders such as dementia (4–7).

This transition is not unique to South Korea but is a global phenomenon, reflecting broader demographic shifts toward older populations worldwide. According to the World Health Organization (WHO), approximately 50 million people are living with dementia globally, with nearly 10 million new cases each year (“Dementia,” 8). This global context underscores the importance of understanding dementia’s impact on individuals and societies alike. The increasing prevalence and the substantial social and economic burden it poses highlight the urgency of addressing dementia care and research on a global scale (“Global action plan on the public health response to dementia 2017–2025,” 9).

Dementia was initially conceptualized following Alois Alzheimer’s 1907 case report of a 51-year-old woman exhibiting rapid memory decline and psychiatric symptoms. Alzheimer’s disease is characterized neurologically by various progressive, fatal symptoms, notably neurofibrillary tangles. Despite posthumous debates over dementia symptoms, it remains a disease characterized by unique pathological phenomena and ongoing neurological deterioration (10). Dementia was initially classified into “presenile” and “senile” based on onset age. However, it was later found that dichotomizing onset age was challenging, with a notably higher frequency observed post-65 years (11–13). Currently, dementia encompasses a range of neurocognitive disorders, including Alzheimer’s, characterized by functional decline due to physical brain changes.

The increase in dementia incidence post-65 years, coupled with the aging trend, has significantly accelerated the rate of individuals with dementia. According to the 2021 South Korea Dementia Status report, the estimated ratio of individuals with dementia among the elderly population aged 65 and over is about 10.3% (840,000 people), an increase from the 9.18% prevalence (540,000 people) reported in 2012. This figure is expected to continue rising, reaching over 3 million (17.2% prevalence) by 2050, highlighting the urgent need for dementia preparedness (14). This trend mirrors global statistics, which project that the number of people living with dementia could reach 152 million by 2050, emphasizing the universal challenge of addressing and managing dementia within aging populations.

The Diagnostic and Statistical Manual of Mental Disorders (DSM)-5 categorizes dementia as a Neurocognitive Disorder (NCD). NCD is further divided into mild and moderate neurocognitive disorders. Cognitive impairment due to brain function damage significantly hinders the ability to perform daily activities (15). According to DSM-5, the cognitive domains impaired in individuals with dementia are classified into six major areas: complex attention, executive function, learning and memory, language, perceptual-motor-visual perception, praxis, and social cognition.

The significance of dementia as a social issue arises from the affected individuals’ inability to perform even simple self-care functions. Dementia has been identified as a decisive factor causing significant differences in Activities of Daily Living (ADL) (16). ADL encompasses basic self-care activities, ranging from “independent performance” to “inability to perform even with assistance.” It typically includes six basic functions developed by Katz et al. (17): bathing, dressing, toilet use, mobility, urinary management, and food intake. The Korean Aging Panel adapted these functions to include “face washing/tooth brushing/hair washing” and “bathing/showering,” reflecting Korean cultural characteristics, thus comprising seven basic functions. Additionally, Instrumental Activities of Daily Living (IADL) measure more complex social and cultural functions.

The correlation between dementia and daily living abilities has been established through numerous studies (18–20). However, most Korean studies have been cross-sectional, lacking sufficient evidence for the longitudinal relationship between ADL levels and dementia. This study aims to first understand the patterns of change in ADL and IADL among the general middle-aged and older population in Korea and how these patterns differ by dementia status. The research questions are as follows:

What are the patterns of change in Activities of Daily Living (ADL) and Instrumental Activities of Daily Living (IADL) among the middle-aged and older population in Korea?

How do these patterns differ by dementia status in the Korean middle-aged and older population?

## Methods

### Research data

The study utilized data from the first (2006) to the eighth (2020) wave of the Korea Longitudinal Study of Ageing (KLoSA). The initial survey in 2006 sampled 10,254 middle-aged and older adults (born before 1961) residing in households across South Korea, excluding Jeju Island. The KLoSA was conducted through face-to-face computer-assisted personal interviewing (CAPI) by interviewers visiting participants. Therefore, it was possible to objectively measure dementia. Additionally, due to the interview-based study design, there were no non-responses in the survey. The survey alternated between a general survey in even years and a thematic survey in odd years. In the fifth survey (2014), an additional sample of 920 individuals born in 1962 and 1963 was included. The ninth basic survey was completed in 2022. The data used in this study included responses from 6,133 participants who consistently responded from the first to the eighth wave (2020), encompassing mild cognitive impairment (*N*=38), non-dementia condition group (*N*=6,004; 97.90%), and dementia group (*N*=91; 1.48%).

### Measurement methods

Activities of Daily Living (ADL) Difficulty Scale: ADL is a scale that assesses basic daily living abilities, including changing clothes, washing/brushing teeth/hair washing, bathing/showering, eating, going outside the room, and using the toilet and controlling bowel and bladder movements. Respondents indicated whether they needed help with these activities, answering ① no help needed, ③ partial help needed, or ⑤ complete help needed. For scoring, needing partial (③) or complete (⑤) help was scored as ‘1’, and not needing help (①) was scored as ‘0’. The total ADL item scores create an ADL index with a maximum score of 7, indicating more problems in daily living abilities. The internal reliability of the ADL index was satisfactory, with values from the first to the seventh wave being.966,.970,.981,.980,.980,.973,.980 respectively.

Instrumental Activities of Daily Living (IADL) Difficulty Scale: IADL assesses the ability to perform instrumental daily activities. It includes grooming, housework, meal preparation, laundry, short trips, using transportation, shopping, managing finances, making and receiving phone calls, and taking medication. Similar to ADL, respondents indicated whether they needed help with these activities. The scoring was the same as for ADL, with a maximum IADL index score of 10. A higher IADL index indicates more problems in performing instrumental daily activities. The internal reliability of the IADL index was also satisfactory, with values from the first to the seventh wave being.951,.956,.965,.964,.963,.962,.970 respectively.

Dementia Onset: In the seventh wave (2018), dementia onset was assessed with three response options: “Yes”, “Mild Cognitive Impairment (MCI)”, and “No”. As shown in **Table 1**, 91 cases (1.5%) answered “Yes”, 38 cases (.6%) answered “Mild Cognitive Impairment”, and 6,004 cases (97.9%) answered “No”. For repeated measures analysis, those who answered “Yes” to dementia onset were classified as “older adults with dementia”, and the rest as “older adults without dementia”. The average age of the dementia group was approximately 13 years older than the non-dementia group (refer to **Table 1**). The evaluation determined if the respondents had ever received a diagnosis of dementia or mild cognitive impairment (MCI) from a doctor.3

**Table 1:** Demographic information of the study participants - 7th year (2018).

Variable	Dementia (Yes)	Mild Cognitive Impairment	Non-Dementia (No)	*F* or χ^2^ (*p*)*	Full Sample
Total	91 (1.5%)	38 (0.6%)	6,004 (97.9%)		10,254 (100%)
Gender				4.712 (0.030)	
- Female	60 (65.9%)	25 (65.8%)	3,485 (58.0%)		3,345 (58.5%)
- Male	31 (34.1%)	13 (34.2%)	2,522 (42.0%)		2,372 (41.5%)
Age	82.36 (SD=7.195)	83.63 (*SD*=7.503)	70.96 (*SD*=9.18)	-14.464 (<.001)	71.21 (*SD*=9.30)
Income	1689.80 (*SD*=1598.64)	1518 (*SD*=1779.19)	2728.14 (*SD*=2417.81)	4.843 (<.001)	2661.35 (*SD*=2403.45)
Education				50.866 (<.001)	
-< Elementary	64 (70.3%)	31 (81.6%)	2550 (42.5%)		2645 (43.1%)
- Middle school	11 (12.1%)	2 (5.3%)	1077 (17.9%)		1090 (17.8%)
- High school	12 (13.2%)	3 (7.9%)	1768 (29.4%)		1783 (29.1%)
- > College	4 (4.4%)	2 (5.3%)	609 (10.1%)		615 (10.0%)

* Difference between dementia and no dementia group.

### Analysis method

The number of dementia cases was 1.50% (*N*=91) of the total analysis sample (*N*=10,254), which was minimal. Therefore, this study analyzed the developmental trajectory of ADL or IADL for all 10,254 cases using Latent Growth Models and compared the ADL, IADL averages between the 6,004 non-dementia condition group and the 91 dementia group, including mild cognitive impairment, using repeated measures ANOVA. According to G power analysis, a minimum of 22 cases per group was required to maintain 0.80 power (1-beta) for repeated measures ANOVA.

Latent Growth Curve Models (LGCM) were used to understand the developmental trajectory of daily living abilities/instrumental daily living abilities for the entire group (including both non-psychosocial and psychosocial disorder response groups). Three models were tested: Linear Model, Quadratic Model, and Cubic Model. The optimal model was selected based on the data-model fit index criteria (RMSEA<.08; CFI>.90; TLI>.90; SRMR<.08) and the parsimonious rule.

The LGCM framework was utilized to model the trajectory of ADL and IADL difficulties over time. In this model, the intercept represents the initial level of difficulty in activities of daily living at the baseline measurement, while the slope factor represents the rate of change in these difficulties over time. We specify the model as follows:


$$Y_{it}=\beta_{0}+\beta_{1}T_{it}+\beta_{2}X_{i}+\epsilon_{it}$$

where *Y_it_* represents the outcome variable (level of difficulty) for individual i at time t, *T_it_* is the time variable, *Xi* represents a vector of covariates, and ϵ*_it_* is the error term.

Covariates included age, gender, income, education level, and presence of chronic conditions to control for their potential confounding effects on the trajectory of ADL difficulties. These variables were selected based on their theoretical and empirical relevance to the study’s outcomes.

Repeated measures ANOVA was conducted to compare the ADL, IADL averages between the non-dementia condition group (including mild cognitive impairment *N*=6,004 + 38) and the dementia group (*N*=91). The within-group variable was the ADL, IADL scores measured five times, and the between-group variable was the diagnosis of dementia. JASP0.17.1 (21) was used for both Latent Growth Model analysis and repeated measures ANOVA. Missing data were addressed using full information maximum likelihood (FIML) imputation for analysis.

Repeated measures ANOVA with covariates was employed to analyze the within-subjects effects of time on ADL and IADL difficulties, offering a detailed assessment of changes over multiple time points. This method is particularly designed to test the null hypothesis that there is no temporal variation in daily living difficulties against the alternative hypothesis of significant change. Unlike traditional ANCOVA, the repeated measures framework incorporates interaction terms between time and key demographic and health-related covariates such as age, gender, income, and education level, which are shown in **Table 1** as potential confounders. This inclusion is crucial for exploring how changes in ADL and IADL difficulties may vary across different subgroups over time. Additionally, given the small number of dementia cases, using a more complex growth model could have been problematic. The repeated measures approach simplifies the analysis and effectively handles the increased variance seen in the subgroup of older adults diagnosed with dementia, whose variance grows with age, thus ensuring a robust analytical strategy.

## Research results

### Descriptive statistics

As shown in **Table 1**, the average age of the participants in the seventh year (2018) was 71.21, with the average age at the start of the survey being 59.21. While 58.5% of all respondents were female, a higher proportion of women were diagnosed with dementia (65.9%) or mild cognitive impairment (65.8%) compared to men (χ^2^= 4.712, *p*= 0.030). In terms of household income, the dementia group had a significantly lower average (1689.80) compared to the non-dementia group (2728.14). There was also a notable difference in education levels, with a higher percentage of dementia-diagnosed individuals having an education of elementary school or less (70.3%) compared to the non-dementia group (42.51%), suggesting a correlation between education level and dementia.

### Overall ADL and IADL developmental trajectories

**Figure 1:**
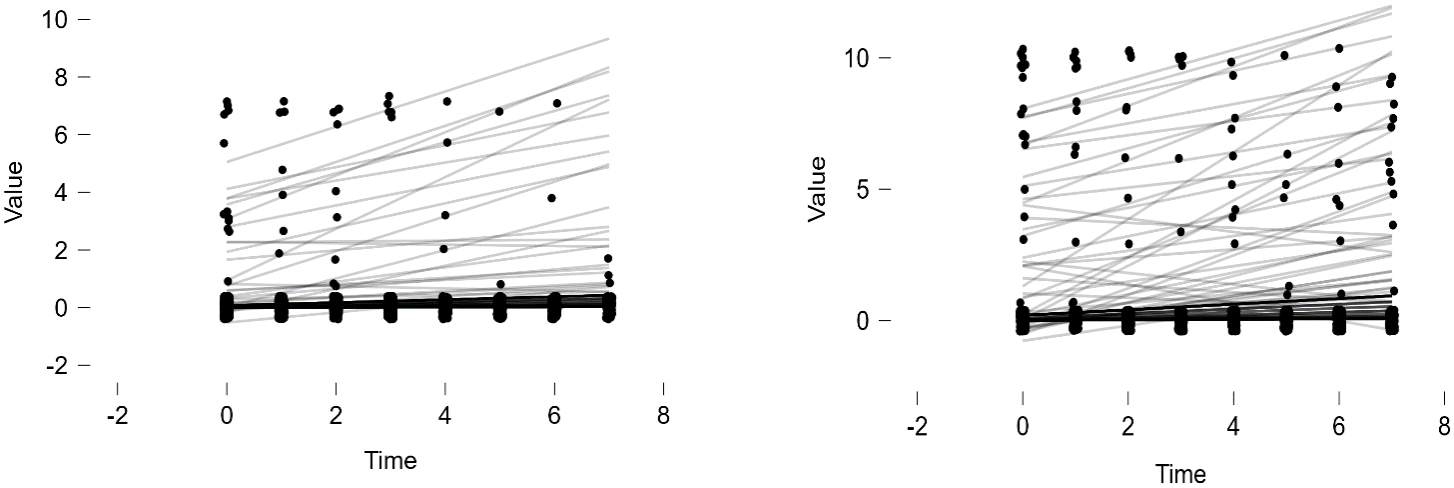
ADL and IADL Index Scores Over Time.

**Figure 2:**
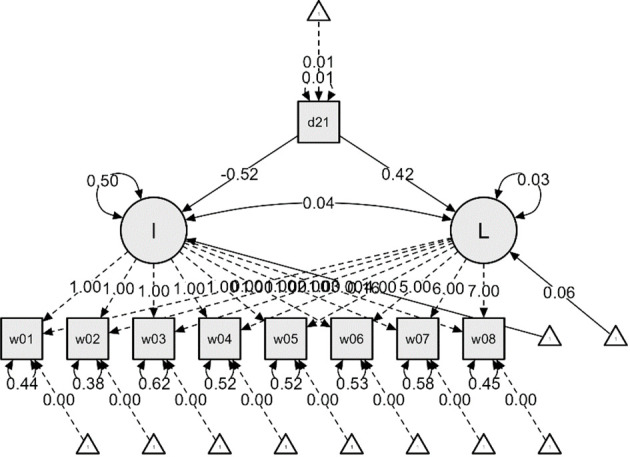
ADL Latent Growth Model Linear Function – From the First Wave (2006) to the Eighth Wave (2020).

Latent Growth Models (Linear Model, Quadratic Model) were tested for the entire group (including both non-dementia and dementia condition groups). The model fit indices for both models are presented in **Table 2**. Among the two models, the Linear Model was deemed appropriate based on the parsimonious rule and the adequacy criterion (CFI>.90) (refer to **Table 2**). In this model, the growth trajectory is seen as curvilinear (refer to **Figures 1**-**2**). As shown in the figures, the scores for ADL and IADL of the participants tended to increase linearly over time, indicating that problems in daily living abilities and instrumental daily living abilities increase with age.

**Table 2:** Model fit indices for IADL latent growth models.

RMSEA	CFI	TLI	SRMR	Chi-square	df
Linear Model*	0.056	0.956	0.957	0.075	1227.189
Quadratic Model	0.040	0.981	0.978	0.031	556.007

The model denoted by an asterisk (*) was selected for this study.

**Figure 3:**
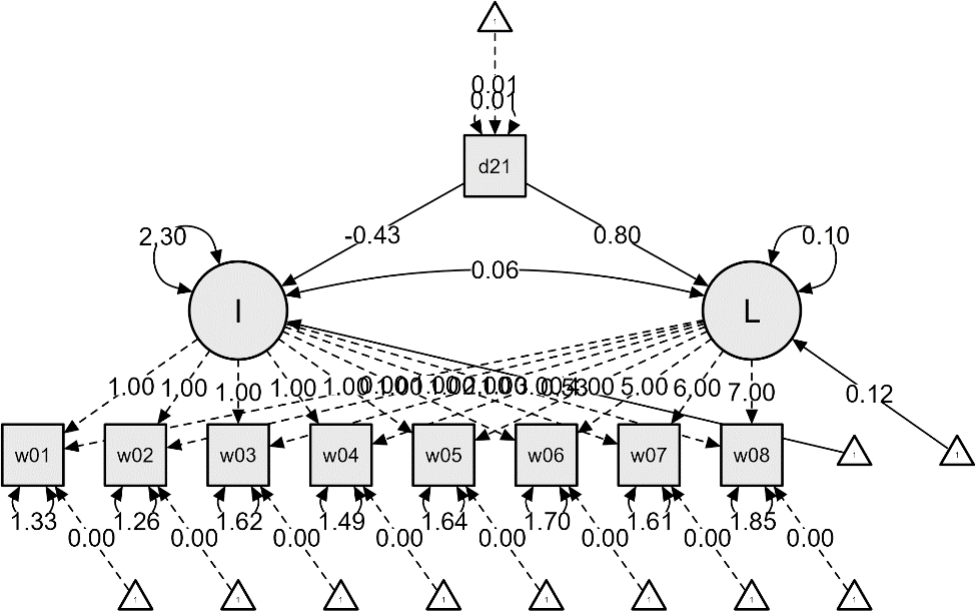
IADL Latent Growth Model Linear Function – From the First Wave (2006) to the Eighth Wave (2020).

Additional analyses were conducted to examine the impact of dementia status on the initial values and growth rates of ADL and IADL, resulting in **Figures 2** and **3**. The effects of dementia status on the initial values of ADL and IADL were -0.52 and -0.43, respectively, indicating slight differences. However, the impact on the growth rates showed a nearly twofold difference, with 0.42 for ADL and 0.80 for IADL. This suggests that respondents diagnosed with dementia experienced a more rapid deterioration in IADL, i.e., instrumental daily living difficulties, compared to ADL.

### Repeated measures ANOVA based on dementia status

**Figure 4:**
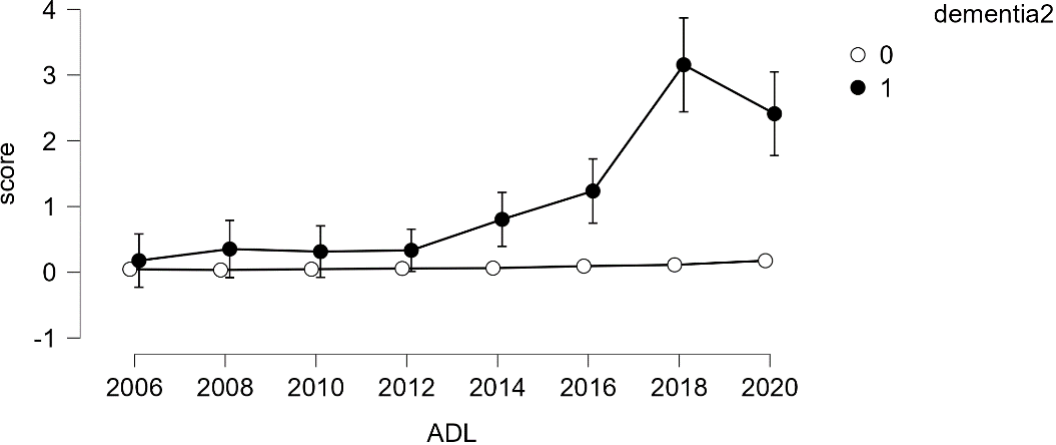
Difficulty in Performing Daily Living Activities – Comparison Between Older Adults with Dementia and Without Dementia.

**Figure 5:**
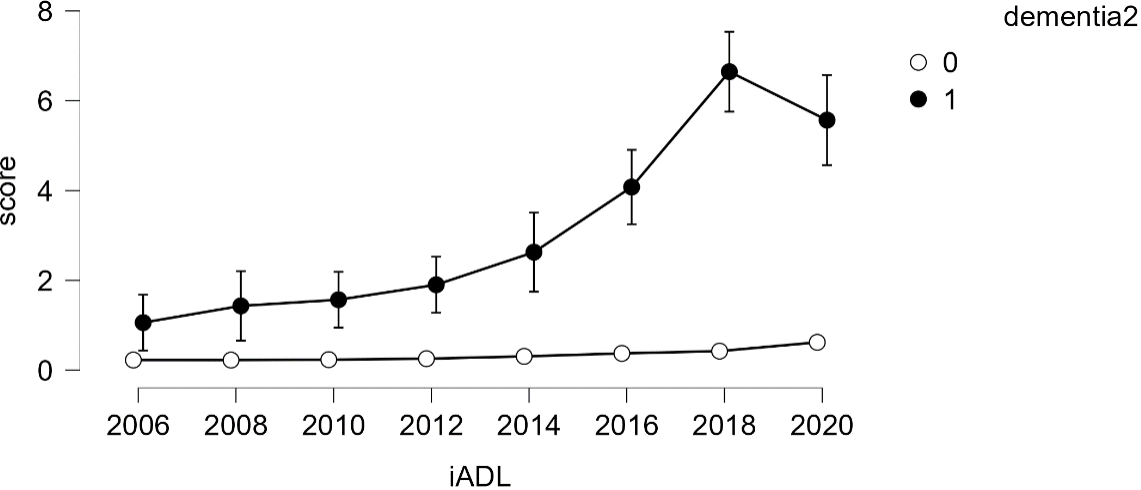
Difficulty in Performing Instrumental Daily Living Activities – Comparison Between Older Adults with Dementia and Without Dementia.

Given the small number of dementia cases (*N*=91), analyzing growth models based on levels of dementia appeared challenging. Therefore, a simpler model, repeated measures analysis, was employed to compare growth between groups. The results indicated that older adults diagnosed with dementia consistently showed an increase in both ADL and IADL scores over time, while those without a dementia diagnosis maintained a score of 0 (refer to **Figures 4**, **5**). Variance was generally higher in the group of older adults diagnosed with dementia and increased with age. This suggests that older adults with dementia experienced a progressive intensification of difficulties in performing daily activities (indicated by higher ADL scores) and greater variability within the group (indicated by the lengthening of the box plots). A similar trend was observed in instrumental daily living difficulties (IADL). Notably, IADL scores were overall higher and showed greater variation, which can be attributed to the broader range of IADL scores (0-10) compared to ADL scores (0-7). Interestingly, the ADL difficulties for the dementia group peaked in 2018 and then slightly decreased in 2020. However, the ADL/IADL scores for the non-dementia group remained consistent during this period.

### Interaction effects between dementia and ADL/IADL

An examination of the interaction effects between dementia and ADL/IADL was conducted (refer to **Table 3**). The sphericity test for within-group effects indicated that the data violated the assumption of sphericity. To compensate, the Greenhouse-Geisser correction was applied, adjusting the degrees of freedom in the analysis. Overall, the repeated measures factor (time) effect was statistically significant (*F*=8.422, *p*<.001). Additionally, the interaction effect between dementia and ADL was also statistically significant (*F*=171.351, *p*<.001), indicating significant changes in ADL over time and differences in ADL changes based on dementia status. This effect difference showed statistical significance between dementia groups (*F*=241.012, *p*<0.001), indicating that regardless of time changes, there were overall differences in ADL based on dementia status.

**Table 3:** Interaction effects between dementia diagnosis and ADL scores over time.

Within Subjects Effects					
Sphericity Test	Sum of Squares	df	Mean Square	F	p
ADL					
- None	17.237^a^	7.000^a^	2.462^a^	8.422^a^	<.001^a^
- Greenhouse-Geisser	17.237	4.647	3.709	8.422	<.001
ADL * Dementia Status					
- None	350.705^a^	7.000^a^	50.101^a^	171.351^a^	<.001^a^
- Greenhouse-Geisser	350.705	4.647	75.469	171.351	<.001
Residual					
- None	10427.969	35665.000	0.292		
- Greenhouse-Geisser	10427.969	23676.637	0.440		
Between-Group Effects					
Cases	Sum of Squares	*df*	Mean Square	*F*	*p*
Dementia	332.749	1	332.749	241.012	<.001
Residual	7034.317	5095	1.381		

Type III Sum of Squares. ^a^ Mauchly’s test indicated violation of sphericity (p<.05).

Similar results were observed for the interaction effects between dementia and IADL (refer to **Table 4**). The sphericity test for within-group effects indicated that the data violated the assumption of sphericity. The Greenhouse-Geisser correction was applied, resulting in a statistically significant effect for the repeated measures factor (time) (*F*=23.755, *p*<.001). The interaction effect between dementia and IADL was also statistically significant (*F*=135.565, *p*<.001), indicating significant changes in IADL over time and differences in IADL changes based on dementia status. This effect difference showed statistical significance between dementia groups (*F*=332.003, *p*<0.001), indicating that regardless of time changes, there were overall differences in IADL based on dementia status.

**Table 4:** Interaction effects between dementia diagnosis and IADL scores over time within subjects effects.

Sphericity Test	Sum of Squares	*df*	Mean Square	*F*	*p*
IADL					
- None	189.258^a^	7.000^a^	27.037^a^	23.755^a^	<.001^a^
- Greenhouse-Geisser	189.258	4.732	39.995	23.755	<.001
IADL * Dementia Status					
- None	1080.039^a^	7.000^a^	154.291^a^	135.565^a^	<.001^a^
- Greenhouse-Geisser	1080.039	4.732	228.239	135.565	<.001
Residual					
- None	40583.788	35658.000	1.138		
- Greenhouse-Geisser	40583.788	24105.127	1.684		
*Between Subjects Effects*					
Cases	Sum of Squares	*df*	Mean Square	*F*	*p*
Dementia	2326.469	1	2326.469	332.003	<.001
Residual	35695.555	5094	7.007		

Type III Sum of Squares. ^a^ Mauchly’s test indicated violation of sphericity (*p*<.05).

## Discussion

This study sought to elucidate the developmental trajectories of Activities of Daily Living (ADL) and Instrumental Activities of Daily Living (IADL) among middle-aged and older adults in South Korea, with a particular focus on variations attributable to the presence of dementia. Utilizing longitudinal data from the Korea Longitudinal Study of Ageing (KLoSA) spanning from 2006 to 2020, this research compared a cohort without dementia (*N*=6,042) to a smaller cohort exhibiting dementia responses (*N*=91).

The findings corroborate existing literature, indicating an inverse relationship between age and the capacity to perform ADL and IADL tasks (1, 2). This decline is notably linear, diverging from the complex patterns of cognitive development often observed in gerontology, where crystallized intelligence may increase with age, in contrast to the decline of fluid intelligence (22). Importantly, this study aligns with and extends findings from recent cognitive research, which suggests a near-linear decline in specific cognitive functions from early adulthood, particularly in areas such as processing speed and executive functions (23).

The analysis further demonstrates that the presence of dementia significantly exacerbates difficulties in IADL more so than in ADL. This disparity is attributed to the higher cognitive and social demands of IADLs, which involve complex interactions and decision-making processes not as prevalent in ADL tasks. This finding underscores the substantial influence of sociocultural factors on the manifestation of dementia, supporting assertions from prior studies (2, 24, 25).

Interestingly, the study identifies ADLs as potentially more indicative of early dementia, highlighted by the “elbow” phenomenon observed in the longitudinal data, where significant increases in ADL difficulty were detected prior to concentrated periods of dementia diagnoses (2016 and 2018). This suggests that even basic ADL tasks might serve as preliminary indicators for dementia screening.

The observed gender disparity in dementia incidence, with a significantly higher percentage of diagnoses among women, underscores the importance of adopting gender-specific research methodologies and healthcare strategies. This finding emphasizes the necessity to delve into the biological, genetic, and sociocultural factors contributing to this disparity, with the aim of refining diagnostic and treatment protocols accordingly. However, the question of gender differences in dementia incidence remains contentious. According to Ruitenberg et al. (26), their large population-based study indicates no gender differences in dementia incidence until advanced age. Notably, after the age of 90, the incidence of Alzheimer’s disease increases for women, while men exhibit higher rates of vascular dementia across all age groups. This complex pattern suggests that further research is essential across diverse contexts and over prolonged periods to fully understand these gender variations. Additionally, the multitude of factors influencing these disparities requires in-depth exploration. Indeed, evidence indicates that risk factors for dementia vary significantly by gender. For instance, midlife hypertension and hypercholesterolemia are known predictors of Alzheimer’s disease later in life for both sexes. However, the rising prevalence of diabetes, which is more notable in women, is linked with a considerable risk of cognitive impairment (27). Additionally, obesity in midlife may pose a higher dementia risk for women than for men. These findings highlight the need for differentiated strategies in the treatment and management of dementia, tailored to gender-specific epidemiological and physiological profiles. Implementing such an approach would not only address the immediate health disparities but also enhance the overall effectiveness of healthcare interventions aimed at reducing the impact of dementia across populations.

The research acknowledges limitations associated with the measurement tools used for ADL and IADL assessments, which are based on simplified self-report survey items. This methodological constraint may lead to an underestimation of the complexity of daily living challenges faced by individuals with dementia, suggesting a need for methodological refinement and the development of more comprehensive assessment tools.

In conclusion, this study contributes significantly to the gerontological literature by providing empirical evidence of the distinct patterns of ADL and IADL difficulties in relation to dementia within a Korean context. It calls for an expansion of the methodological toolkit used in aging studies, emphasizing the integration of technological advancements and nuanced survey instruments. By addressing the noted limitations and adopting a culturally sensitive approach, future research can more effectively tailor interventions and policies to meet the diverse needs of aging populations globally. This study not only enriches our understanding of dementia’s impact on daily activities but also serves as a crucial step toward improving the quality of life for older adults through targeted and effective healthcare strategies.

## Data availability statement

The original contributions presented in the study are included in the article/supplementary materials. Further inquiries can be directed to the corresponding author.

## Ethics statement

Ethical approval was waived for the study involving humans in accordance with the local legislation and institutional requirements. Written informed consent to participate in this study was not required from the participants or the participants’ legal guardians/next of kin in accordance with the national legislation and the institutional requirements. The Korea Longitudinal Study of Ageing (KLoSA) used in this research was officially approved by the Statistics Office (Approval Number 33602). Furthermore, the KLoSA is de-identified data that does not contain personal identifiers such as names and social security numbers.

## Author contributions

SC: Writing – original draft, Writing – review & editing.
